# Supplementary material for: Short-term molecular and cellular effects of ischemia/reperfusion on vascularized lymph node flaps in rats
Source: PLoS One. 2020 Oct 6;15(10):e0239517. doi: 10.1371/journal.pone.0239517 (PMC7537894; doi:10.1371/journal.pone.0239517)
Supplement: S1 Raw images — (PDF) [file pone.0239517.s001.pdf]

S1_raw_images

Short-term molecular and cellular effects of ischemia/reperfusion on vascularized lymph node flaps in rats

Florian S. Frueh[1,2*], Bijan Jelvani[1], Claudia Scheuer[1], Christina Körbel[1], Bong-Sung Kim[2], Nicole Lindenblatt[2], Pietro Giovanoli[2], Yves Harder[3], Emmanuel Ampofo[1], Michael D. Menger[1] and Matthias W. Laschke[1]

[1]Institute for Clinical and Experimental Surgery, Saarland University, 66421 Homburg/Saar, Germany

[2]Department of Plastic Surgery and Hand Surgery, University Hospital Zurich, University of Zurich, 8091 Zurich, Switzerland

[3]Division of Plastic, Reconstructive and Aesthetic Surgery, Ospedale Regionale di Lugano, Ente Ospedaliero Cantonale, 6900 Lugano, Switzerland

[*] **Corresponding Author**

Florian S. Frueh, MD PhD

Division of Plastic Surgery and Hand Surgery

University Hospital Zurich

Rämistrasse 100

8091 Zurich

Switzerland.

Phone: +41 (0)44 255 11 11

E-mail: florian.frueh@usz.ch

Lymph node

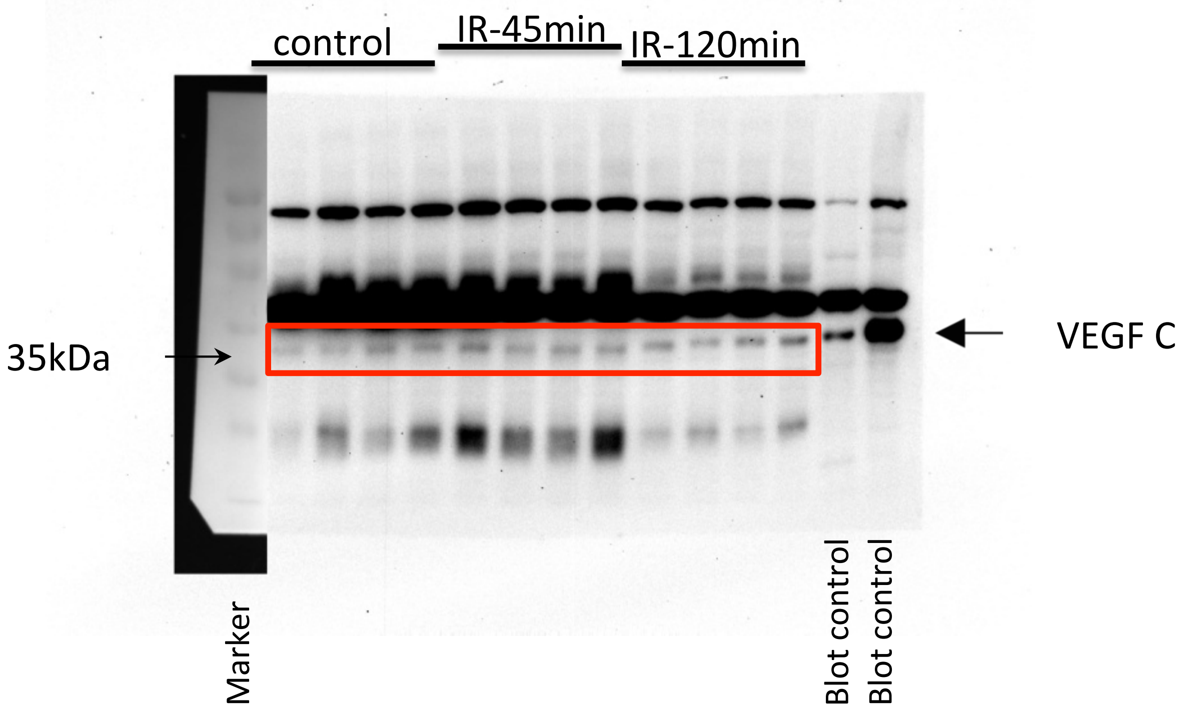

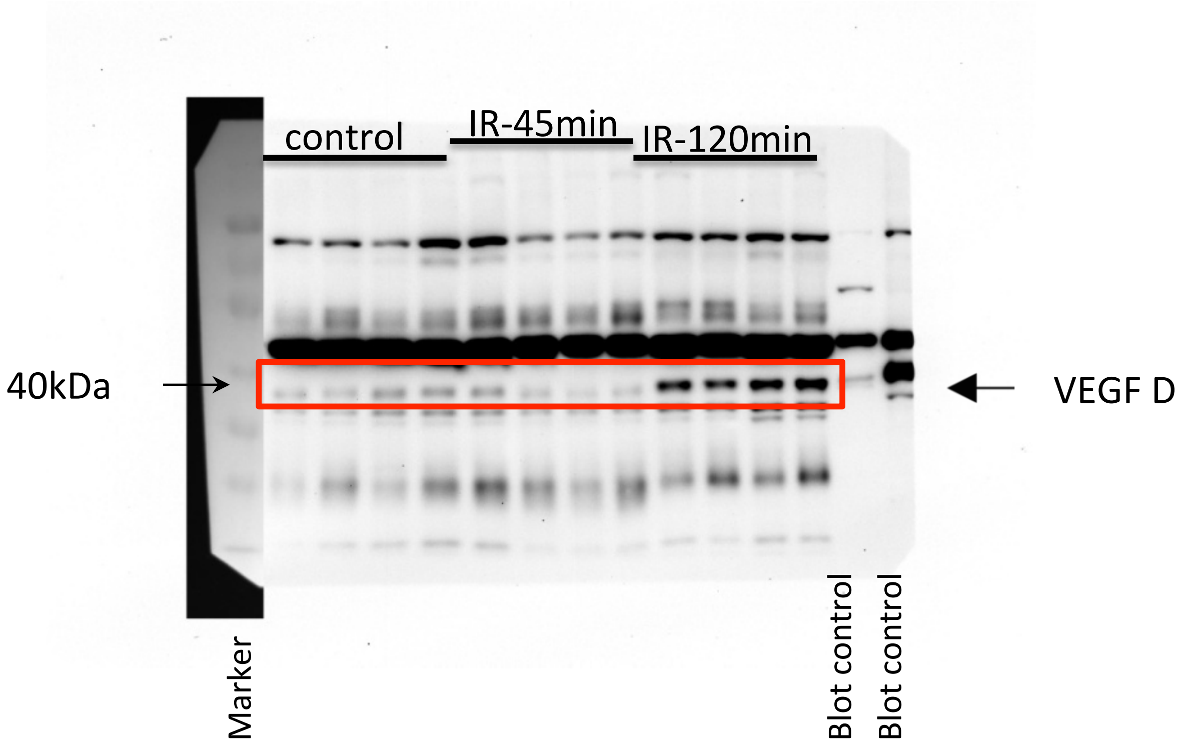

Uncropped images of blots presented in Fig. 5A

Lymph node

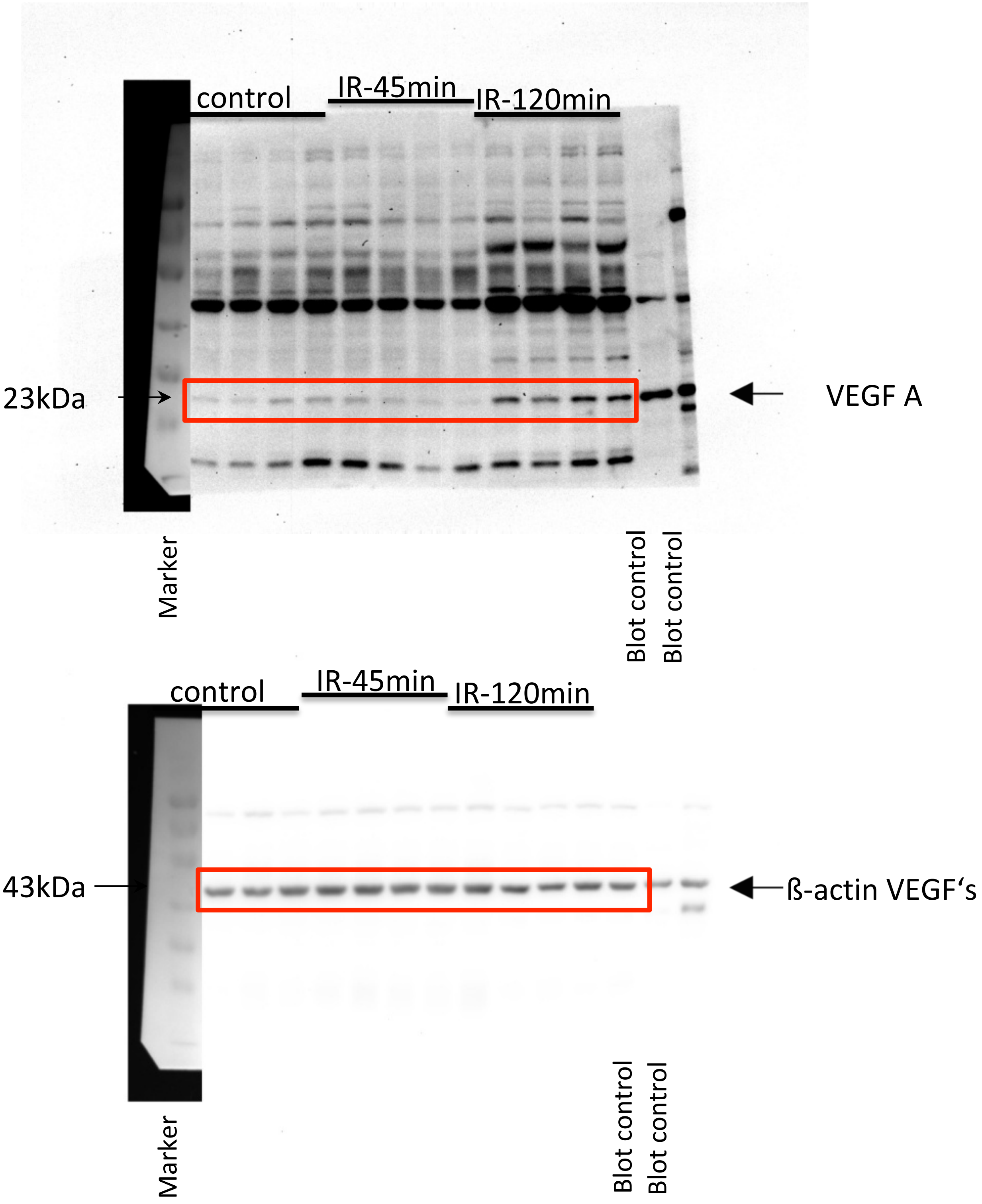

Uncropped images of blots presented in Fig. 5A

Adipose tissue

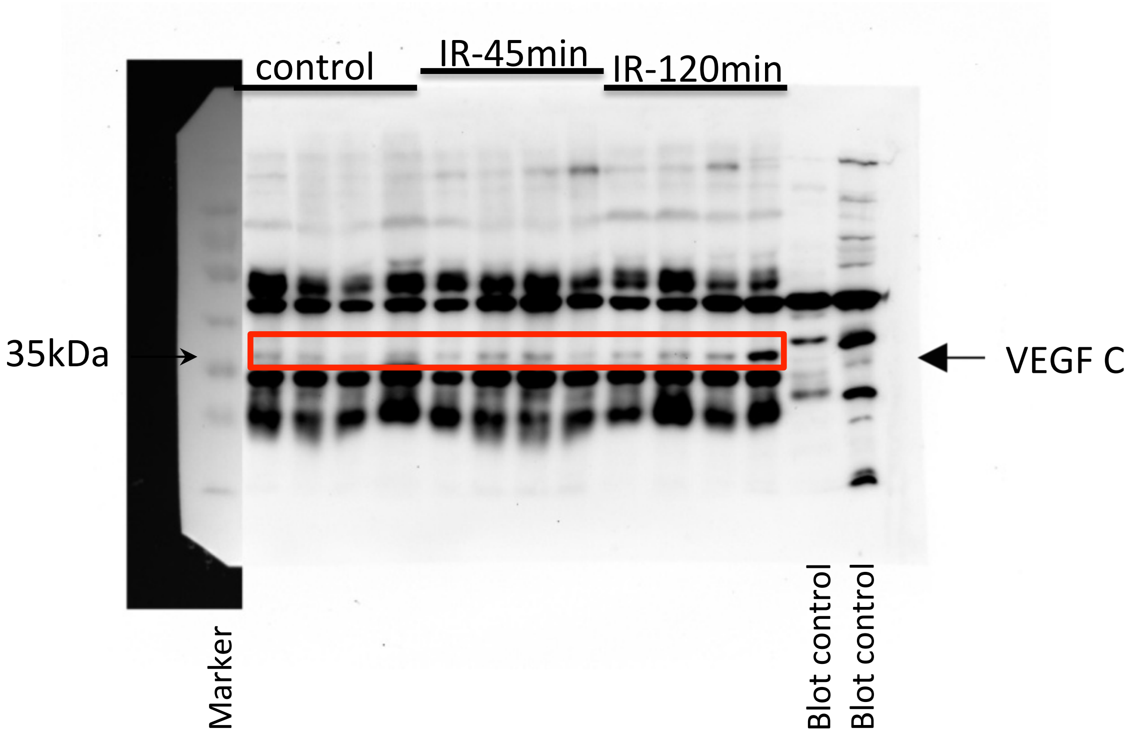

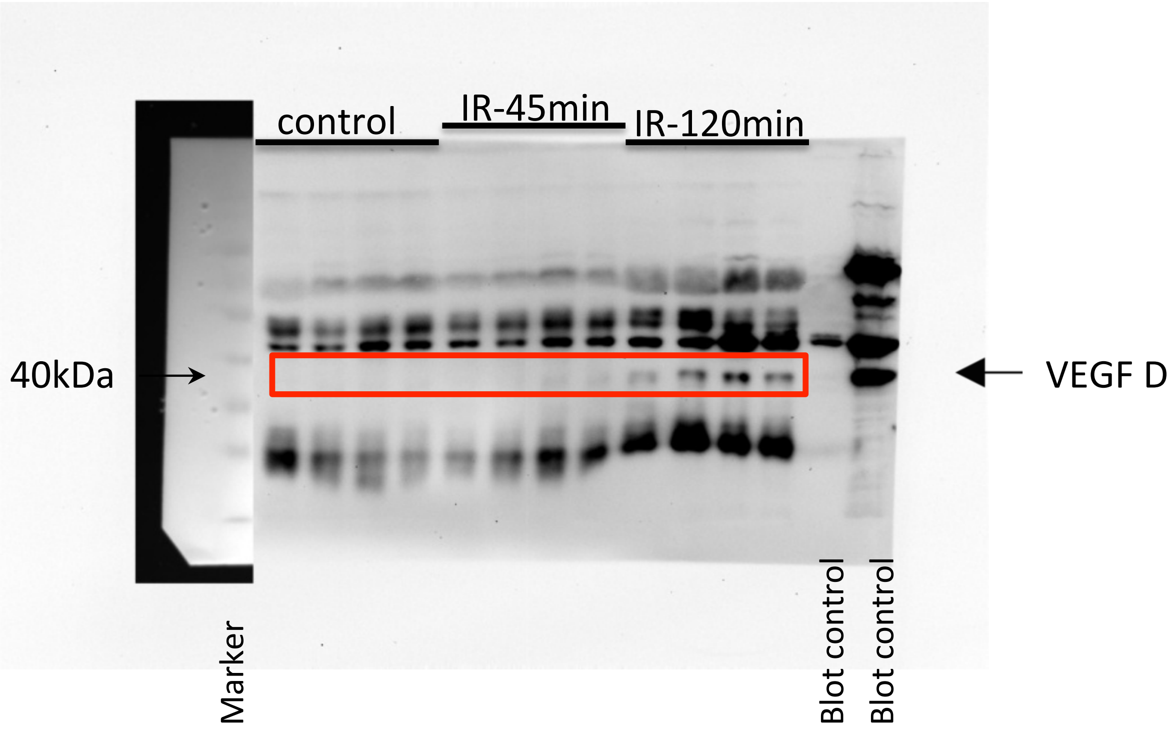

Uncropped images of blots presented in Fig. 5B

Adipose tissue

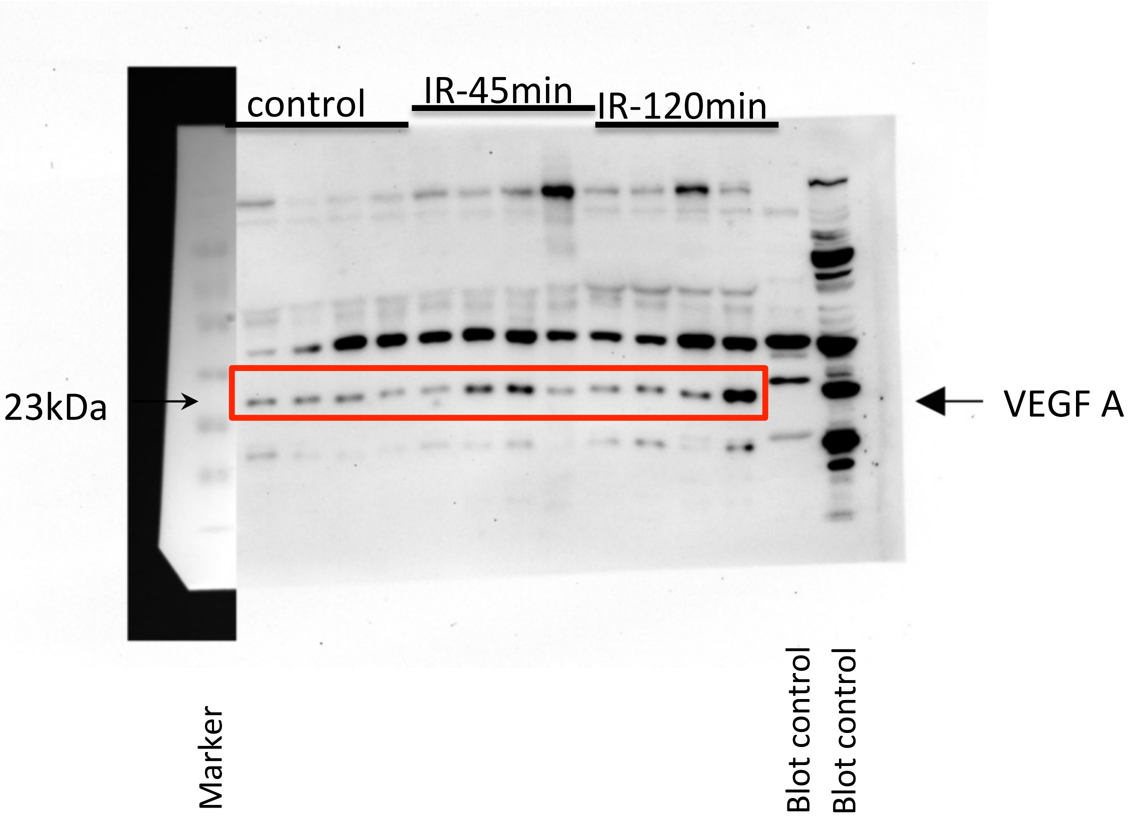

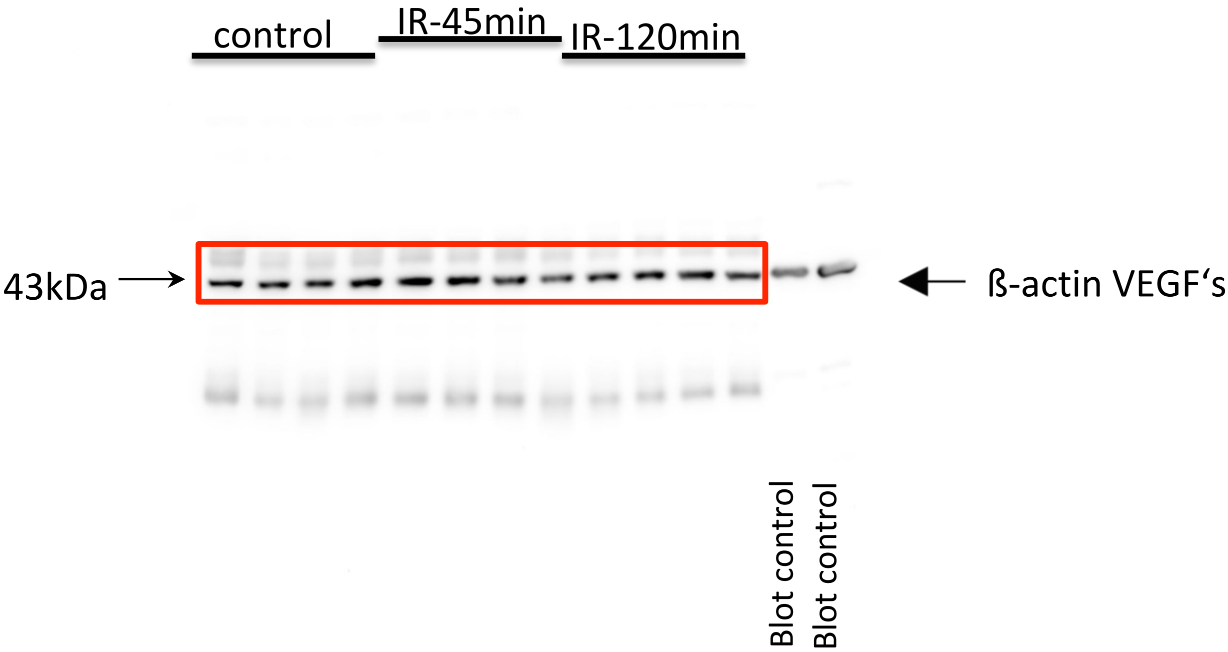

Uncropped images of blots presented in Fig. 5B

Lymph node

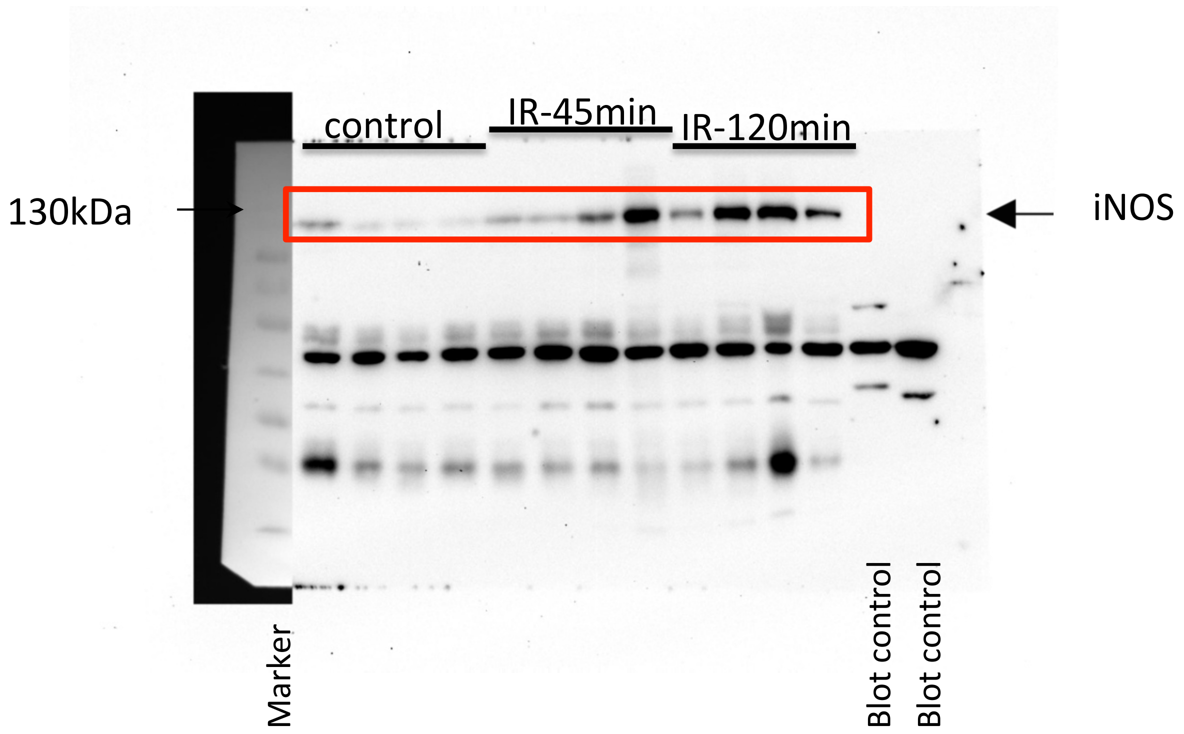

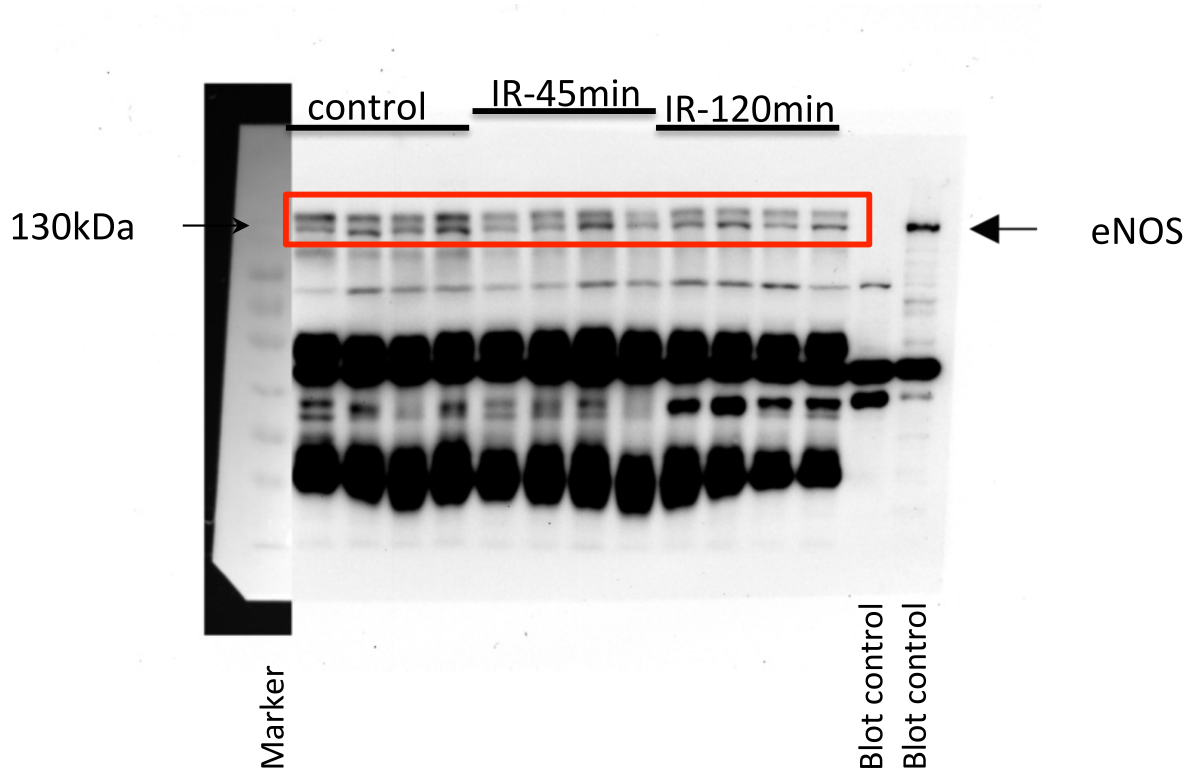

Uncropped images of blots presented in Fig. 5E

Lymph node

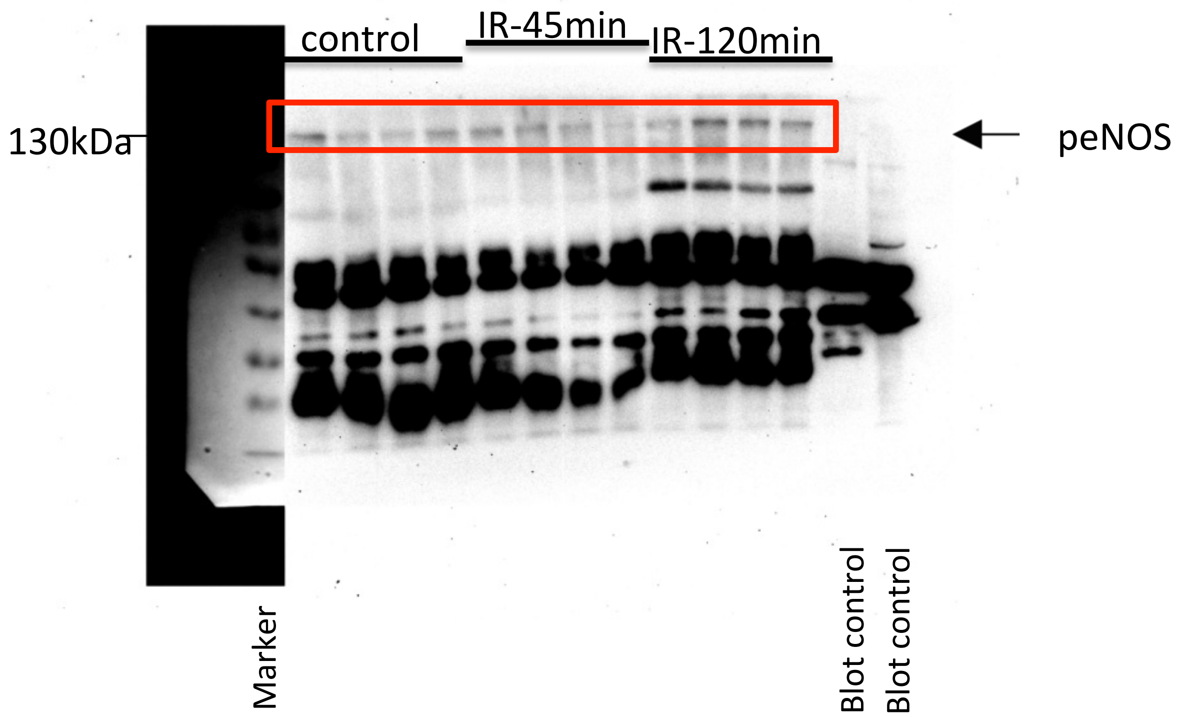

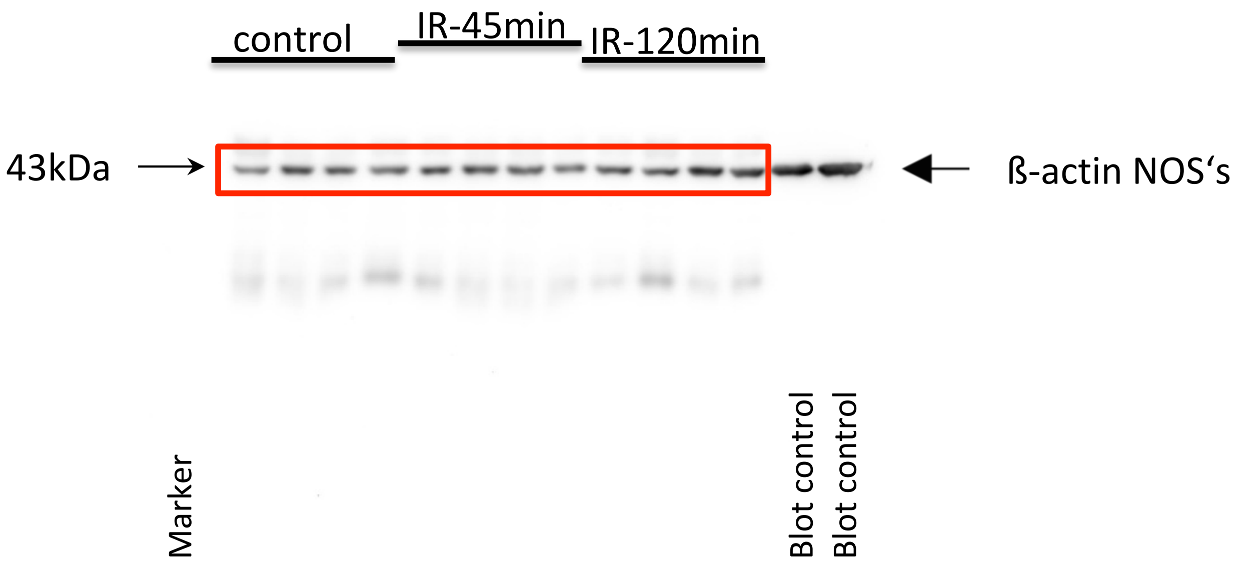

Uncropped images of blots presented in Fig. 5E

Adipose tissue

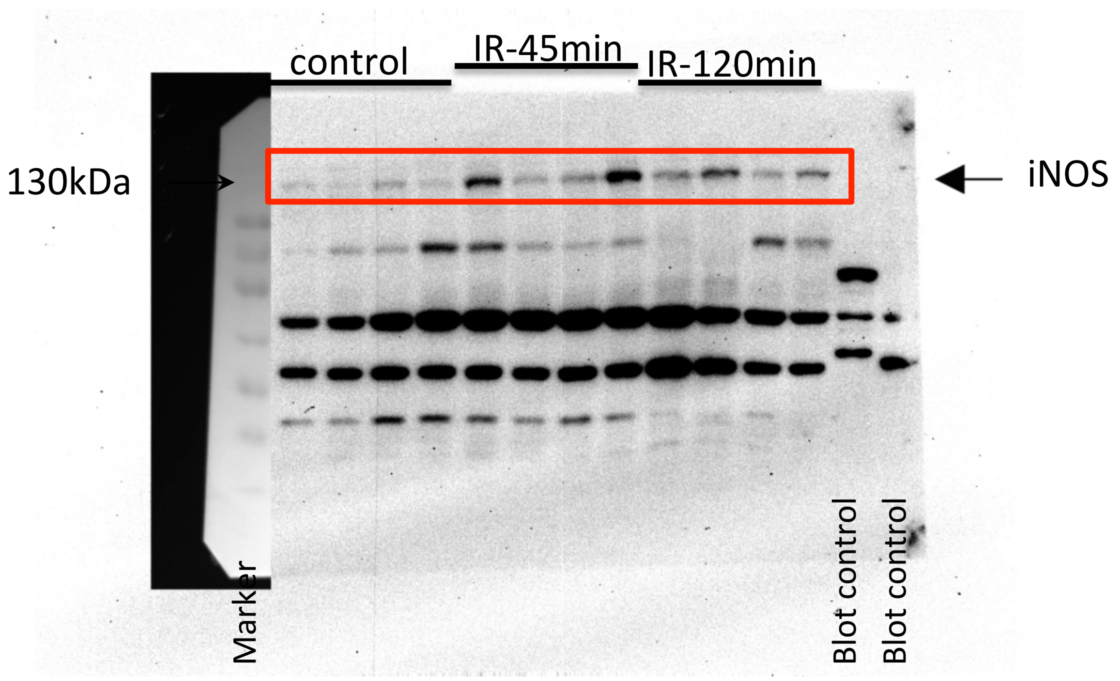

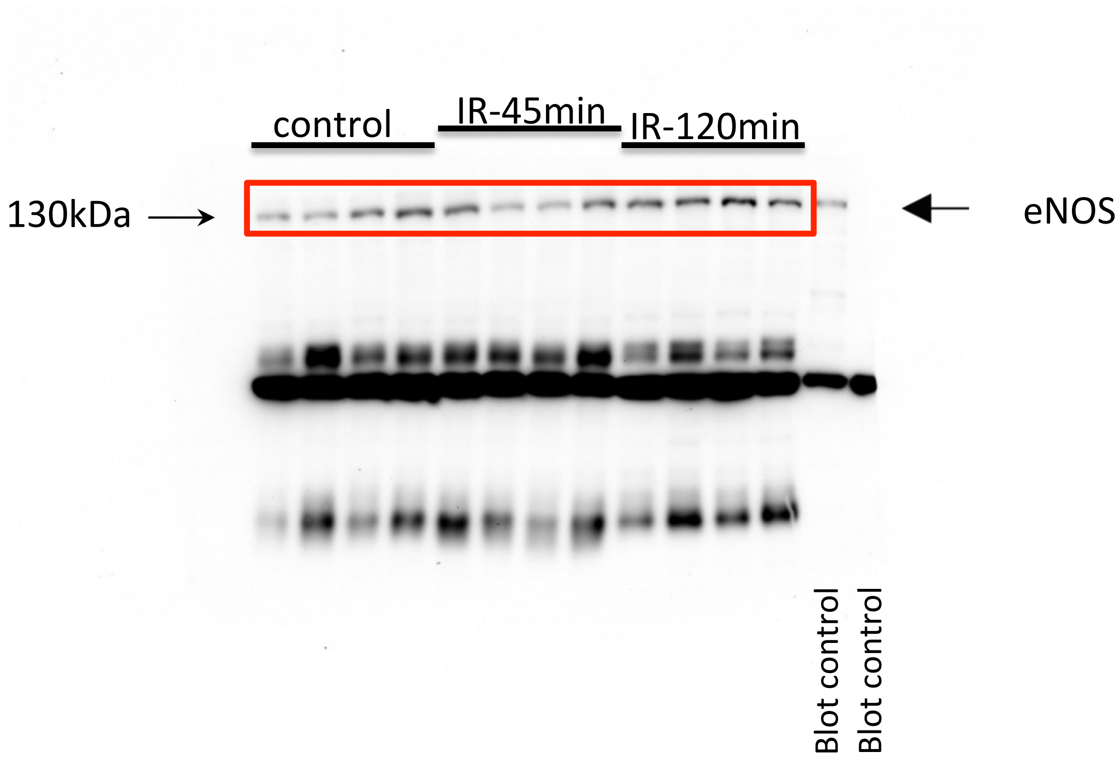

Uncropped images of blots presented in Fig. 5F

Adipose tissue

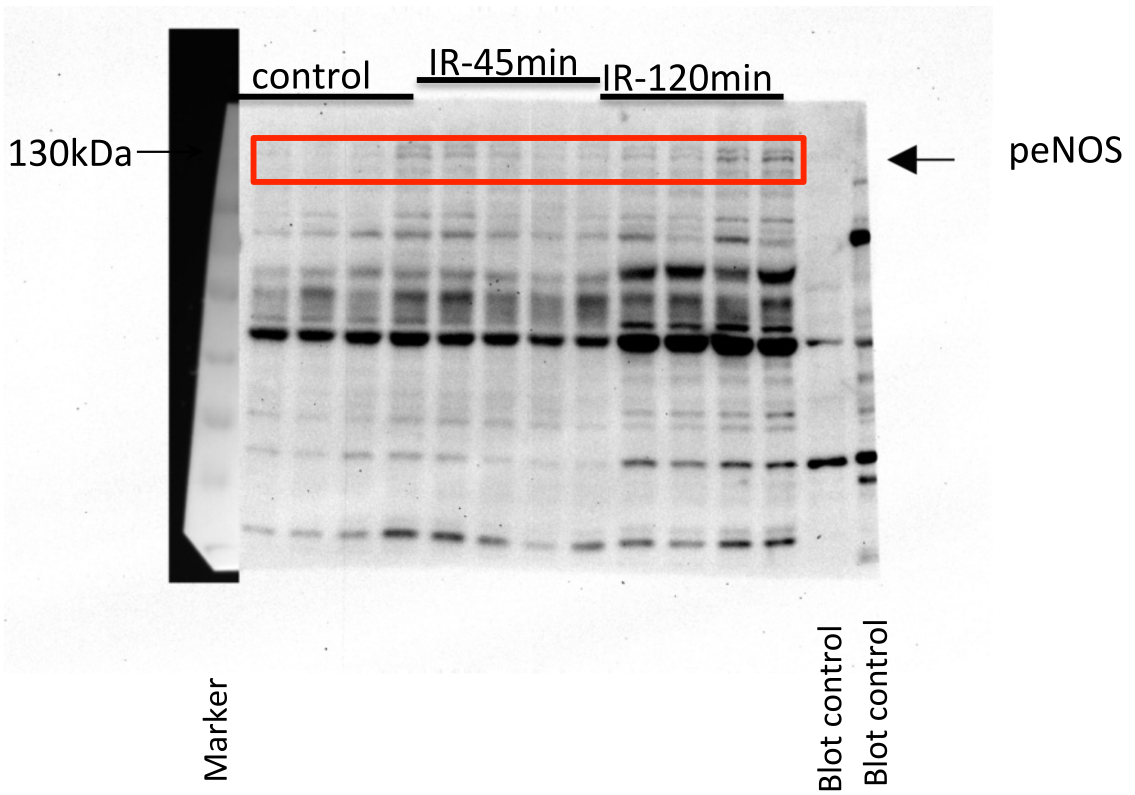

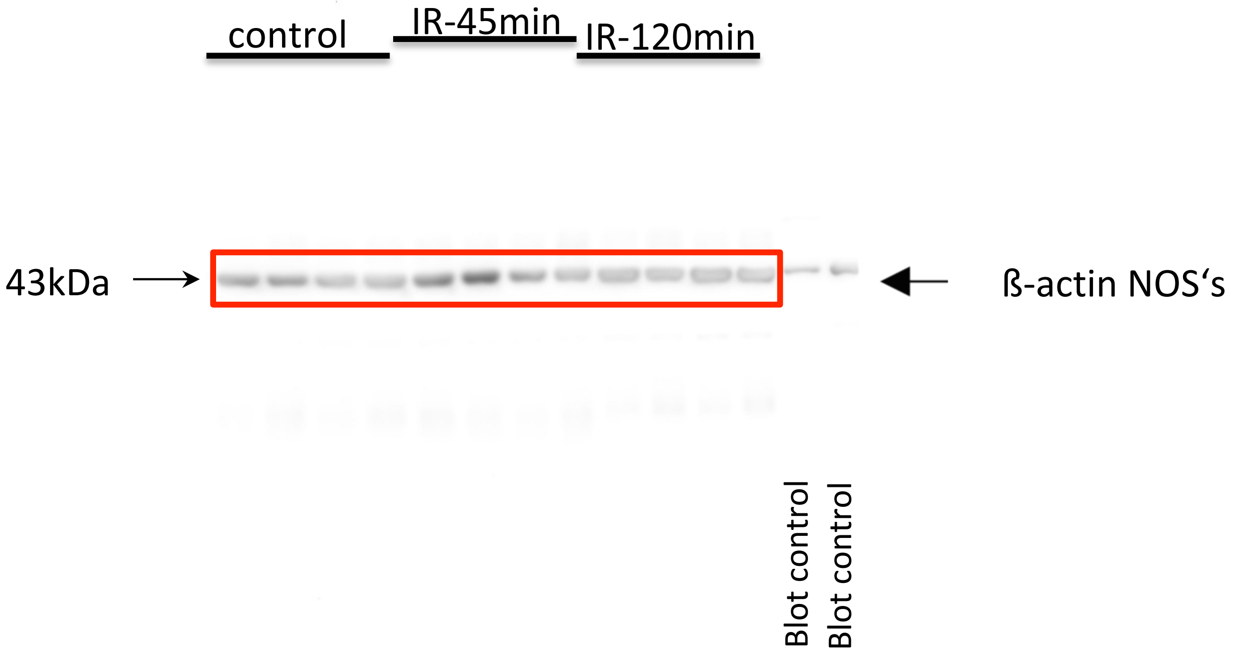

Uncropped images of blots presented in Fig. 5F
