# Supplementary material for: Short-term molecular and cellular effects of ischemia/reperfusion on vascularized lymph node flaps in rats
Source: PLoS One. 2020 Oct 6;15(10):e0239517. doi: 10.1371/journal.pone.0239517 (PMC7537894; doi:10.1371/journal.pone.0239517)
Supplement: Attachment — Submitted filename: Response to reviewers.docx [file pone.0239517.s002.docx]

**Review of the manuscript PONE-D-19-21979 by Frueh et al.**

**Reply to the comments of reviewer 1**

*Reviewer #1: The authors evaluated the molecular and cellular effects of ischemia/reperfusion on vascularized lymph node flap in a rat model. After ischemia treatment of 45 min or 120 min and followed by reperfusion for 24 hours, the oxygen content, level of oxidative stress and growth factors were examined. The authors separate lymph nodes and perinodal adipose tissue to observe the ischemia/reperfusion effects. The number of proliferating and apoptotic cells were increased after ischemia/reperfusion treatment. Some data still required to be added to make the manuscript more complete.*

We thank the reviewer for the fair and constructive comments. Please find our point-by-point reply in the following.

Specific comments:

*Reviewer comment 1*

*In Fig 3, the authors observed the expression of LYVE. Did any changes in the expression of LYVE under different ischemia time or in the location of LYVE expression? Any scientific relevant of using LYVE in this staining, since the authors only observed the structure of the lymph node under ischemia treatment.*

We thank the reviewer for this important comment. The reviewer is correct that LYVE-1 staining in this part of our investigation was only used for descriptive purposes and not for a quantitative analysis of lymphatic vessel density. In this context it should be noted that the expression of LYVE-1 in the lymphatic sinuses of lymph nodes is not limited to lymphatic endothelial cells but is also observed in other cell types, such as reticular cells (Bai et al., 2011). Consequently, a quantification of the lymphatic vascular network (ie, the sinuses) within individual lymph nodes is not appropriate using LYVE-1 as marker.

**Reference**

Bai Y, Wu B, Terada N, Ohno N, Saitoh S, Saitoh Y, Ohno S. Histological study and LYVE-1 immunolocalization of mouse mesenteric lymph nodes with "in vivo cryotechnique". Acta Histochem Cytochem. 2011;44:81-90.

*Reviewer comment 2*

*The results on Fig. 4 presented the increase level of VEGF-A. Did the authors stain for CD31 or other angiogenesis markers to evaluate whether the blood vessel density were also increased in lymph nodes or adipose tissue?*

According to the comment of the reviewer, we have performed additional immunohistochemical stainings of VLN flaps using the endothelial cell marker CD31 to quantify the microvessel density (mm^-2^) within individual lymph nodes and the perinodal adipose tissue. Interestingly, lymph nodes undergoing 45 and 120 minutes of ischemia exhibited a significantly reduced microvessel density when compared to non-ischemic controls. In contrast, the microvascular network of the perinodal adipose tissue was less sensitive to ischemia/reperfusion injury. This novel information is now included in the revised version of our manuscript (see Figure 4; Results, page 6/7, lines 171-188 as well as Discussion page 12/13, lines 341-347; marked in yellow)

*Reviewer comment 3*

*The increase number of proliferating cells were identified in Fig. 5 by using PCNA+ marker. However, the other results indicated the cellularity were decreased after 45 min/120 min ischemia and 24 hours reperfusion treatment (in Fig. 3). What kind of cells were the proliferating cells? And what kind of cell were the caspas 3 labeled?*

The reviewer is correct that despite an overall reduction of cellularity, we observed an increasing number of proliferating cells in the lymph nodes of ischemic VLN flaps. However, this is a common finding after ischemia/reperfusion injury. Due to technical issues, we were not able to further characterize PCNA^+^ and caspase-3^+^ cells with co-stainings using lymphocyte-specific antibodies. Instead, we have performed a morphological analysis of individual PCNA^+^ and caspase-3^+^ cells within ischemic lymph nodes and found that the majority of them were lymphocytes. This is in line with the findings of a recently published study by Perrault et al. (2020), who reported a particularly high sensitivity of the immune cell component of lymph nodes to ischemia reperfusion injury. This novel information is now included in the revised version of our manuscript (see Results, page 9, lines 234-236; marked in yellow).

**Reference**

Perrault DP, Lee GK, Bouz A, Sung C, Yu R, Pourmoussa AJ, Park SY, Kim GH, Jiao W, Patel KM, Hong YK, Wong AK. Ischemia and reperfusion injury in superficial inferior epigastric artery-based vascularized lymph node flaps. PLoS One. 2020;15:e0227599.

*Reviewer comment 4*

*How the authors choose the 45 and 120 min as the ischemia time and 24 hours as the reperfusion time?*

We chose the ischemia time based on realistic clinical scenarios. In fact, 45 minutes represent an uncomplicated microvascular VLN flap transplantation and 120 minutes represent a difficult or suboptimal microvascular procedure. The reperfusion time was chosen based on our previous experience with ischemia/reperfusion experiments and it has recently been shown that shorter reperfusion times may reduce the biological reaction to ischemia of VLN flaps (Perrault et al., 2020).

**Reference**

Perrault DP, Lee GK, Bouz A, Sung C, Yu R, Pourmoussa AJ, Park SY, Kim GH, Jiao W, Patel KM, Hong YK, Wong AK. Ischemia and reperfusion injury in superficial inferior epigastric artery-based vascularized lymph node flaps. PLoS One. 2020;15:e0227599.

*Reviewer comment 5*

*In the Discussion, the authors emphasized on the different effects after ischemia treatment on lymph nodes and adipose tissue. However, the ischemia effect on lymph nodes were presented in the Fig. 4. There were no cell cellularity, HO-1+, PCNA+ or immune cell examination on adipose tissue.*

According to the comment of the reviewer, we have performed an additional immunohistochemical analysis of the perinodal adipose tissue, evaluating the fraction of HO-1^+^, PCNA^+^ and caspase-3^+^ cells as well as the number of infiltrating CD68^+^ macrophages and MPO^+^ neutrophilic granulocytes. Of interest, we found that the cells within the perinodal adipose tissue were not markedly affected by 45 or 120 minutes of ischemia. This indicates again a higher resistance of adipose tissue to ischemia/reperfusion injury when compared to the lymph node component of VLN flaps. This novel information is now included in the revised version of our manuscript (Figure 7; see Results, page 9, lines 236-239 and lines 247-250 as well as page 10, lines 267-281; see Discussion, page 13/14, lines 369-374; marked in yellow).

Two new co-authors from the Division of Plastic Surgery and Hand Surgery, University Hospital Zurich and from the Institute for Clinical & Experimental Surgery, Saarland University, markedly contributed to our novel immunohistochemical analyses. Accordingly, both co-workers have been included in the author list of the revised manuscript (see page 1, lines 6 and 7; see page 23, lines 632 and 636; marked in yellow).
