# Supplementary material for: Short-term molecular and cellular effects of ischemia/reperfusion on vascularized lymph node flaps in rats
Source: PLoS One. 2020 Oct 6;15(10):e0239517. doi: 10.1371/journal.pone.0239517 (PMC7537894; doi:10.1371/journal.pone.0239517)
Supplement: Attachment — Submitted filename: Response to reviewers.docx [file pone.0239517.s003.docx]

**Review of the manuscript PONE-D-19-21979R1 by Frueh et al.**

We have appreciated the fair and constructive comments of the reviewers. Please find our point-by-point reply in the following.

**Reply to the comments of reviewer 1**

*Reviewer comment 1:*

*In the result section (page 5), “r and t = 900 μm“, and in the figure legend of Fig. 2, “R and T= 900 μm” are not compatible.*

We have corrected the corresponding section according to the reviewer’s comment (see page 6, line 151, marked in yellow).

*Reviewer comment 2:*

*On page 6, “ In the IR-120 group, however, we observed a partial loss of the lymph node architecture (Figs. 3F and 3G)”, which particular part was lost?*

Our statement was only based on qualitative assessments of lymph node morphology. However, we did not further analyze whether specific parts of the lymph node architecture were lost. Accordingly, we have removed this vague statement from our revised manuscript and now solely focus on the quantitative analysis of lymph node cellularity (see page 6, lines 156-159 and page 12, line 306-312; marked in yellow).

*Reviewer comment 3:*

*In Fig. 4D, the CD 31 staining seems more abundant by compared to the other two groups, which was inconsistent to the results of Fig. 4 E.*

The reviewer is correct that the CD31 staining in Fig. 4D looks more abundant. However, the red area in the center of the lymph node is due to local hemorrhages (ie, red stained clusters of erythrocytes). Importantly, microvessels characterized by a CD31^+^ endothelium are marked with arrowheads for the reader. In addition, the hemorrhages are now marked with separate arrows (see revised Fig. 4D and page 7, line 190, marked in yellow).

*Reviewer comment 4:*

*According to the discussion, authors referenced Gust’s paper indicated “an active role of adipose tissue in driving the inflammatory response after IR injury [26]. The overexpression of stress and inflammatory markers as well as inflammatory cell infiltration of mature adipose tissue following IR injury. But other authors found that severe ischemia is associated with loss of mature adipocytes, which are replaced with new adipocytes derived from resident adipose-derived progenitor cells [27]. “*

*Since the results showing a higher level of eNOS presented in the perinodal adipose tissue in Fig. 6, there was no significance cell proliferation, apoptosis, no oxidative stress in Fig. 7, which were inconsistent with the results from the reference that the authors provided. How to explain these findings?*

We appreciate this important comment. Indeed, it is noteworthy that in our investigation, the adipose tissue was characterized by a less dramatic reaction to IR injury. However, a more thorough look at the referenced animal model by Gust et al. reveals important technical differences when compared to our experimental model. Importantly, Gust et al. used an IR model with magnetic skin compression. In contrast, our IR model exposes the adipose tissue to indirect ischemia without mechanical manipulation. Hence, the less extensive tissue trauma might also result in fewer IR-associated tissue alterations. We now provide this explanation in the discussion section of our revised manuscript (see page 13-14, lines 365-373, marked in yellow).

*Reviewer comment 5:*

*The results indicated an increasing level of VEGF-A after ischemia condition (Fig. 5). The results were not consistent with that in Fig. 4, in which the micro-vessel density was decreased after ischemia condition.*

From a biological point of view, this finding is not surprising because IR-related tissue damage leads to the loss of microvascular structures and local hypoxia. Consequently, angiogenic growth factors such as VEGF-A are upregulated, promoting angiogenic neovessel growth. However, angiogenesis is a time-consuming process with a slow growth rate of new microvessels of 5 µm per hour. Hence, 24 hours of reperfusion may be too short to demonstrate a subsequent increased vascularization. This information is now provided in the revised discussion of our manuscript (see page 13, lines 345-352 and page 23, lines 627-628; marked in yellow).

**New reference**

Utzinger U, Baggett B, Weiss JA, Hoying JB, Edgar LT. Large-scale time series microscopy of neovessel growth during angiogenesis. Angiogenesis. 2015;18:219-232.

*Reviewer comment 6:*

*The staining of CD31 in Fig. 4D was not compatible with Figs. 4E and 4F.*

See reply to comment 4.

**Reply to the comments of reviewer 2**

*Reviewer comment 1:*

*Indicate in the title, in the abstract and when the objective of the study is stated at the end of the introduction, that the main goal is to investigate the “immediate” or “initial” changes at the cellular and molecular level after performing lymph node transfers, since the study ends at 24h postop. Findings may be interpreted as biomarkers to identify the initial phase of I/R injury in lymph nodes and perinodal tissue.*

We thank the reviewer for this important comment. According to his suggestion, we now state in the title, abstract and introduction that our study investigates the *short-term* molecular and cellular effects of I/R on VLN (see page 1, line 3 and page 2, line 41 and page 4, line 99 and page 11, line 296, marked in yellow).

*Reviewer comment 2:*

*The abstract does not indicate which animal model or which lymph nodes are studied, nor the number of animals, nor their distribution in the study groups. Please modify it accordingly.*

According to this comment of the reviewer, we now provide the total number of animals used in the study as well as more details on the animal model in the abstract (see page 2, lines 42-44, marked in yellow).

However, to enhance the readability of the manuscript, we prefer not to mention the distribution of the animals in the study groups in the abstract. In fact, this essential information is already provided in two sections of our manuscript (ie, experimental protocol and figure legends).

*Reviewer comment 3:*

*Please modify the image Figure 1.B as it is not clearly seen.*

We have modified the figure including a more detailed explanation in the corresponding figure legend (see revised Figs. 1B and 1C and corresponding figure legend, page 4, lines 119-124, marked in yellow).

*Reviewer comment 4:*

*In the discussion please focus more on your actual results and their interpretation. This section partially sounds like an introduction. You may move parts to the introduction if needed.*

According to this comment of the reviewer, we have moved parts of the discussion to the introduction and focused more on our results (see page 3, lines 87-94 and pages 11-14, lines 306-385; marked in yellow).

*Reviewer comment 5:*

*In the final paragraph of the discussion (conclusion) try to be more specific and summarize the significant results you have obtained in this study. This will help other researchers and readers to get the most relevant results of this study.*

According to this comment, we have modified the last paragraph of the discussion in our revised manuscript (see page 14-15, lines 398-406, marked in yellow).

*Reviewer comment 6:*

*It is important that readers keep in mind the limitations of this study. Include a summary of the main ones and explain them in the discussion. Examples:*

*- There are numerous growth factors that were not included in the analysis, in this sense, we should not exclude the importance of other biomarkers.*

*- Vascularized lymph node transfers are performed using microsurgical anastomoses, however in this study, VLNTs have been simulated by clamping the vessels.*

*- The current literature indicates that the critical ischemia time in lymph node transfers is 4h, however in this study a shorter ischemic insult has been studied.*

We thank the reviewer for this comment. According to his suggestion, we have included a novel paragraph on the limitations of our study in the discussion section of our revised manuscript (see page 14, lines 386-397, marked in yellow).

*Reviewer comment 7:*

*In the Material and Methods section, it is indicated that an injectable anesthetic protocol (Ketamine and xylazine) is used during surgery, but during the ultrasound, photoacoustic imaging and stereomicroscopy evaluation, inhalation anesthesia with isoflurane is used. Why have you used different protocols?*

The reviewer is correct that flap dissection was performed under intraperitoneal anesthesia using ketamine and xylazine. After transfer and fixation of the animals in the ultrasound/photoacoustic imaging system, maintenance of anesthesia was performed using isoflurane inhalation under continuous monitoring of heart and breathing rate as well as body temperature. On the one hand, inhalation anesthesia is suitable to precisely control the depth of anesthesia. On the other, the animal is fixed in a complex imaging system, which renders intraperitoneal injection of anesthetics cumbersome and a source of bias due to animal manipulation, particularly during photoacoustic imaging.

*Reviewer comment 8*

*In order to strictly adhere to the three R's principle, why have you not used the 8 rats that were used for photoacoustic validation for histology, immunohistochemistry or Western blot studies?*

We thank the reviewer for this important comment. However, it can be answered by maximal standardization of the perioperative protocol. Including these rats in the groups for histology, immunohistochemistry or Western blot analyses would not only have resulted in a different protocol (ie, transfer and delay for photoacoustic imaging), it would also be biased by different anesthesia medications as correctly stated by the reviewer in comment 7. Finally, we performed photoacoustic imaging for the IR-45 and IR-120 groups but not for the control group. Hence, inclusion of these animals for subsequent histological, immunohistochemical or Western blot analyses would have also resulted in uneven numbers of the experimental groups.
