# Supplementary material for: Short-term molecular and cellular effects of ischemia/reperfusion on vascularized lymph node flaps in rats
Source: PLoS One. 2020 Oct 6;15(10):e0239517. doi: 10.1371/journal.pone.0239517 (PMC7537894; doi:10.1371/journal.pone.0239517)
Supplement: Attachment — Submitted filename: Response to reviewers.docx [file pone.0239517.s004.docx]

**Review of the manuscript PONE-D-19-21979R2 by Frueh et al.**

We have appreciated the fair and constructive comments of the reviewer. Please find our point-by-point reply in the following.

**Reply to the comments of reviewer 1**

*Reviewer comment 1*

*In Fig 3, any difference on LYVE expression, since it’s the marker for lymphatic network.*

We thank the reviewer for this comment. However, we have already discussed this point in the first review round and hereby provide our original reply:

“The reviewer is correct that LYVE-1 staining in this part of our investigation was only used for descriptive purposes and not for a quantitative analysis of lymphatic vessel density. In this context it should be noted that the expression of LYVE-1 in the lymphatic sinuses of lymph nodes is not limited to lymphatic endothelial cells but is also observed in other cell types, such as reticular cells (Bai et al., 2011). Consequently, a quantification of the lymphatic vascular network (ie, the sinuses) within individual lymph nodes is not appropriate using LYVE-1 as marker.”

**Reference**

Bai Y, Wu B, Terada N, Ohno N, Saitoh S, Saitoh Y, Ohno S. Histological study and LYVE-1 immunolocalization of mouse mesenteric lymph nodes with "in vivo cryotechnique". Acta Histochem Cytochem. 2011;44:81-90.

*Reviewer comment 2*

*In Fig.4, it will be more convicting when the images of CD31staining in perinodal adipose are provided.*

According to this comment of the reviewer, we now provide immunofluorescent images illustrating the CD31^+^ microvascular network in the perinodal adipose tissue in Fig. 4 (see revised Fig. 4 as well as the corresponding figure legend on page 7, lines 187-190 in the revised version of our manuscript, marked in yellow).
